# An Insulin-Like Growth Factor in *Rhodnius prolixus* Is Involved in Post-feeding Nutrient Balance and Growth

**DOI:** 10.3389/fnins.2016.00566

**Published:** 2016-12-09

**Authors:** Marina S. Defferrari, Ian Orchard, Angela B. Lange

**Affiliations:** Department of Biology, University of Toronto MississaugaMississauga, ON, Canada

**Keywords:** development, RNAi, insect, Rhopr-IGF, metabolism, lipids, carbohydrates

## Abstract

Growth of organisms is modulated by the availability of nutrients and energy, and is mostly regulated by insulin-like growth factors (IGFs) through the insulin signaling system. In insects, IGFs produced by the fat body induce cell division during the molt cycle, regulate adult body size, and have metabolic effects. Here, we describe an IGF from the hematophagous hemipteran *Rhodnius prolixus* and show its activity in regulating growth and metabolism in the post-feeding period during the fifth, and last, nymphal instar. Rhopr-IGF transcript is present in a variety of tissues, with greatest expression in the fat body, the dorsal vessel, and the CNS. We silenced the expression of the transcript using RNA interference, and at 2 weeks after feeding, insects with reduced Rhopr-IGF expression showed increased hemolymph lipid and carbohydrate levels when compared to controls, but no differences were observed in fat body lipid or carbohydrate content. In order to assess the role of Rhopr-IGF in post-feeding growth, double stranded IGF-injected insects were followed through ecdysis, and this treatment resulted in shorter adults, with shorter and narrower wings, when compared to controls. The results suggest that Rhopr-IGF modulates growth in *R. prolixus* most likely through altering the usage of nutrients that are available in the hemolymph.

## Introduction

Insulin, insulin-like growth factors (IGFs), and insulin-like peptides (ILPs) are hormones involved in a variety of physiological processes that signal through a conserved cascade in vertebrates and invertebrates (Badisco et al., 2013; Nässel et al., 2015; Nässel and Vanden Broeck, 2016). They function as nutrient-sensing pathways that regulate metabolism, growth, and development (Badisco et al., 2013; Huimei et al., 2016). Insulin consists of two peptide chains, A and B, connected by two disulfide bonds and stabilized by a third disulfide bond located in the A-Chain. In the pancreas of vertebrates, insulin is processed via the action of prohormone convertases that cleave the propeptide, yeilding insulin along with a third chain of amino acids, the C-peptide (Steiner et al., 2009). IGFs are single-chain polypeptides with structural homology to proinsulin. IGFs regulate the proliferation and differentiation of various cell types and are capable of exerting insulin-like metabolic effects, but unlike insulin, IGFs are produced by most tissues/organs in the body, especially the liver (Cohick and Clemmons, 1993).

A large number of ILPs have been identified in insect species, and their genes are mostly expressed in neurosecretory cells in the brain, such as the insulin-producing cells in *Drosophila* (IPCs) (Rulifson et al., 2002; Nässel et al., 2013; Nässel and Vanden Broeck, 2016). Nonetheless, there are ILPs expressed in other tissues and some of these have been characterized as IGFs, displaying similar sequence and structure to vertebrate IGFs. In *Bombyx mori*, an IGF-like peptide (BIGFLP) was shown to be produced in the pupal fat body in response to 20-hydroxyecdysone (20-E) and to influence the growth of tissues *in vitro* (Okamoto et al., 2009a). The *Drosophila* ILP6 (DILP6) has been characterized as an IGF-like peptide, and is also expressed in the fat body in response to 20-E, and regulates post-feeding growth and development during non-feeding states (Okamoto et al., 2009b; Slaidina et al., 2009). The insect fat body displays analogous functions to the vertebrate liver and adipose tissue, such as controlling the synthesis, storage and utilization of energy reserves (Arrese and Soulages, 2010). In *Drosophila*, oenocytes working in association with the fat body regulate larval growth and feeding by altering lipid metabolism while functioning similarly to vertebrate liver hepatocytes (Gutierrez et al., 2007).

*Rhodnius prolixus* is a hematophagous hemipteran that requires gorging on a blood meal to initiate growth and development into the next instar or adult. We have recently characterized the first ILP from *R. prolixus*, which is expressed only in medial neurosecretory cells in the brain and is involved in the metabolism of lipids and carbohydrates during post-feeding and starvation periods (Defferrari et al., 2016). Here, we describe the presence of another ILP in this same insect, expressed in the central nervous system (CNS) but also in peripheral tissues such as the fat body, dorsal vessel and hindgut. This peptide displays sequence and predicted structure similarities to IGFs and is involved in metabolism and development in a post-feeding stage of *R. prolixus*.

## Materials and methods

### Insects

The *R. prolixus* colony was maintained under 50% humidity at 25°C, and insects were fed on defibrinated rabbit blood (Cedarlane Laboratories Inc., Burlington, ON, Canada) once per instar.

### Identification of Rhopr-IGF

Seven *Drosophila* ILPs (accession numbers NP_648361.1, NP_524012.1, NP_996037.1, NP_648360.2, NP_648359.1, NP_570070.1, NP_570000.1), the computationally predicted *R. prolixus* ILPs (Ons et al., 2011; Mesquita et al., 2015), and the recently described *R. prolixus* ILP, Rhopr-ILP (Defferrari et al., 2016), were used for screening the predicted *R. prolixus* peptidome (available at https://www.vectorbase.org) using the program Geneious 8.1.7 (Kearse et al., 2012). The putative amino acid sequence was identified and its corresponding mRNA sequence was used as a template for designing primers and for subsequently cloning the transcript.

### Rhopr-IGF transcript cloning

Total RNA was extracted using a PureLink® RNA mini kit (Life Technologies Corporation, Carlsbad, CA, USA) from the CNS and the fat body of fifth instar *R. prolixus*. For the amplification of the 5′ and 3′ ends of the Rhopr-IGF transcript, cDNA from each of the tissues was synthesized using a RACE PCR kit (Roche Applied Science, Mannheim, Germany) in combination with gene-specific primers (GSPs) (Supplementary Table 1). The final PCR products were cloned using the pGEM-T Easy Vector system (Promega, Madison, WI, USA) and the clones expressing the desired products were sequenced at the Center for Applied Genomics, at the Hospital for Sick Children in Toronto (MaRS Centre, Toronto, ON, Canada).

The complete coding sequence of Rhopr-IGF was isolated as follows: total RNA was extracted from the CNS and the fat body of fifth instars and cDNA was synthesized using the High Capacity cDNA Reverse Transcription Kit (Applied-Biosystems, Fisher Scientific, Toronto, ON, Canada). PCR was performed combining each of the cDNAs with GSPs (Supplementary Table 1) and the products were cloned using the pGEM-T Easy Vector system (Promega, Madison, WI, USA). The clones expressing the desired products were sequenced at the Center for Applied Genomics, at the Hospital for Sick Children (Toronto, ON, Canada). Sequences were confirmed from at least three independent clones from each tissue to ensure base accuracy.

### Rhopr-IGF sequence analyses

The exon-exon boundaries within the Rhopr-IGF transcript were first predicted with a local BLASTN search against the *R. prolixus* genome (Mesquita et al., 2015), using the program Geneious 8.1.7 (Kearse et al., 2012), where the cDNA sequence of Rhopr-IGF was compared to the genomic sequence in order to predict location and size of exons. The splice regions were confirmed on the platform Berkeley *Drosophila* Genome Project (http://www.fruitfly.org/seq_tools/splice.html) by using the online tool Splice Site Prediction. The sequence was also analyzed for a potential signal peptide and for subcellular localization, using SignalP 4.0 (http://www.cbs.dtu.dk/services/SignalP) and TargetP 1.1 (http://www.cbs.dtu.dk/services/TargetP), respectively. The predicted protein sequence of Rhopr-IGF (KX185519) was aligned with 12 ILP and IGF precursor peptides from different insects and three human insulin and IGF precursor peptides using the program MUSCLE 3.8 (Multiple Sequence Comparison by Log-Expectation—http://www.ebi.ac.uk/Tools/msa/muscle).

### Rhopr-IGF expression analysis by real time quantitative PCR

The spatial expression profile of Rhopr-IGF was quantified using different tissues from unfed fifth instars. RNA was isolated using a PureLink® RNA mini kit (Life Technologies Corporation, Carlsbad, CA, USA) and cDNA was synthesized using 250 ng of RNA from each tissue using the High Capacity cDNA Reverse Transcription Kit (Applied-Biosystems, Fisher Scientific, Toronto, ON, Canada). The cDNAs were individually diluted to the appropriate concentrations and 5 ng of template was used per well for the real time qPCR reactions. Primers for the amplification of Rhopr-IGF (Supplementary Table 1) were designed to amplify fragments of similar size across all experimental and reference genes (rp49, β-actin, and α-tubulin) (Paluzzi and O'Donnell, 2012; Defferrari et al., 2014). The qPCR reactions were carried out on a CFX384 Touch™ Real-Time PCR Detection System (Bio-Rad, Mississauga, ON, Canada) and the expression levels were quantified using the ΔΔCt method (Pfaffl, 2001). Experiments were repeated for a total of three biological replicates with three technical replicates each.

### Temporal analysis of Rhopr-IGF expression by real time qPCR

In order to evaluate any changes in the expression of Rhopr-IGF transcript throughout the fifth instar, insects were dissected at different time points under nuclease-free PBS and RNA was extracted as described above. Insects were dissected immediately and 1 week after ecdysis into fifth instars. Two weeks after ecdysis, a group of 30 insects was fed on defibrinated rabbit blood and tissues (CNS, dorsal vessel and fat body) were dissected at 3 h, 1, 3, 7, 14, and 21 days after feeding. Insects dissected at 21 days after feeding had already started ecdysis into adults but had not yet ecdysed. The expression was quantified by real time qPCR as described above. Five insects were dissected at each time point and the experiment was repeated for a total of three biological replicates.

### Double-stranded RNA synthesis

A double-stranded RNA fragment consisting of 560 base pairs from the coding region of Rhopr-IGF transcript (dsIGF) was synthesized using the T7 Ribomax Express RNAi System (Promega, Madison, WI, USA), following the manufacturer instructions, combined with GSPs conjugated with the T7 RNA polymerase promoter (Supplementary Table 1). A dsRNA fragment of the ampicillin resistance gene (dsARG) from the pGEM-T Easy Vector system (Promega, Madison, WI, USA) was used as negative control throughout the experiments (Lee et al., 2013; Defferrari et al., 2014). Rhopr-ILP dsRNA was produced as previously described (Defferrari et al., 2016).

### Injections of double-stranded RNA into fifth instar *R. prolixus* and transcript expression knockdown analysis

Injections of dsIGF and dsARG were performed using with a Hamilton syringe of 10 μL volume capacity (Hamilton Company, Reno, NV, USA) into the hemocoel of fifth instar *R. prolixus* 1 week after the insects had been fed on a blood meal. Insects were divided into two groups of 20–25 individuals: (1) dsIGF-injected and (2) control dsARG-injected. For the quantification of Rhopr-IGF expression knockdown, the CNS, the fat body and the dorsal vessel from each of the two groups were dissected on day 2 and 7 post-injection. The knockdown was analyzed through quantitative PCR, as described above, and Rhopr-IGF transcript expression in dsIGF-injected insects was quantified relative to the expression of the transcript in dsARG-injected insects.

In order to investigate possible changes in Rhopr-IGF expression after Rhopr-ILP knockdown, and in Rhopr-ILP expression after Rhopr-IGF knockdown, a third group of 10 insects was injected with the dsRNA targeting the Rhopr-ILP transcript. The CNS and the fat body of such insects were dissected on day 7-post-injection and the expression of Rhopr-IGF was analyzed. The expression of Rhopr-ILP was quantified in the CNS of dsIGF-injected insects that were dissected on day 7-post-injection. As described above, the expression of the transcripts was analyzed by quantitative PCR, relative to the expression of dsARG-injected insects.

### Hemolymph collection from Rhopr-IGF knockdown insects

One week after the injection of the dsRNA, the fed fifth instars were immobilized and 5 μL of hemolymph were collected from the cut end of a leg, using a 10 μL capacity micro-pipette. The hemolymph was placed in 50 μL of 10% cold TCA and the samples were centrifuged for 10 min at 20°C and 8000 g. The supernatants were transfered to new tubes and both pellets and supernatants were kept for subsequent measurements of lipids (as lipoproteins) and carbohydrates (including both glucose and trehalose), respectively. The pellets were resuspended in 200 μL of isopropanol for the lipid measurements.

### Collection of the fat body from Rhopr-IGF knockdown insects

One week after the dsRNAs were injected into the insects, the fat body sheet lining the ventral abdominal segments was removed from fed fifth instars under *R. prolixus* physiological saline (NaCl 150 mM, KCl 8.6 mM, CaCl_2_ 2.0 mM, MgCl_2_ 8.5 mM, NaHCO_3_ 4.0 mM, glucose 34.0 mM, HEPES 5.0 mM, pH 7.0). The fat body was placed in either 200 μL isopropanol or 200 μL 10% cold TCA, sonicated for two cycles of 3 s each and centrifuged for 10 min at 20°C and 8000 g. Forty microliter of the supernatants were transfered to new tubes and kept for the measurements of total lipid (isopropanol) or carbohydrate content (10% TCA) in the fat body.

### Measurements of hemolymph lipid level and fat body lipid content

The lipids associated with lipoproteins in the hemolymph (pellets of TCA precipitated samples), and the lipid content in the fat body (isopropanol extracted fat body) of Rhopr-IGF knockdowns and dsARG-injected insects, were measured as previously described (Patel et al., 2014).

### Hemolymph and fat body carbohydrate measurements

The hemolymph and fat body carbohydrates were measured as previously described (Defferrari et al., 2016). For the measurement of carbohydrates, 40 μL of the supernatant (after TCA precipitation of protein) was used from either hemolymph or fat body samples.

### Analyses of body and wing size, and body weight of Rhopr-IGF knockdown insects after ecdysing into adults

The fed fifth instars that were injected with dsIGF or dsARG were allowed to ecdyse into adults and 2–3 days after ecdysing the following measurements were taken: (1) body length, (2) body width, at the widest part of the abdomen, (3) wing length, (4) wing width, at the widest part of each wing, and (5) body weight. The experiment was repeated three times with 4–6 insects per group each time.

### Statistical analyses

Results are shown as means plus standard errors. The significance of differences between means of dsIGF-injected and dsARG-injected groups was determined using Student's *t*-test. The difference between each time point and the time point used as reference in the temporal analysis of Rhopr-IGF expression was also determined using Student's *t*-test. Results were considered statistically different when *P* < 0.05. All analyses were carried out using the program SigmaPlot (Systat Software, San Jose, California, USA).

## Results

### Characterization of Rhopr-IGF transcript and its predicted amino acid sequence

**Figure 1:**
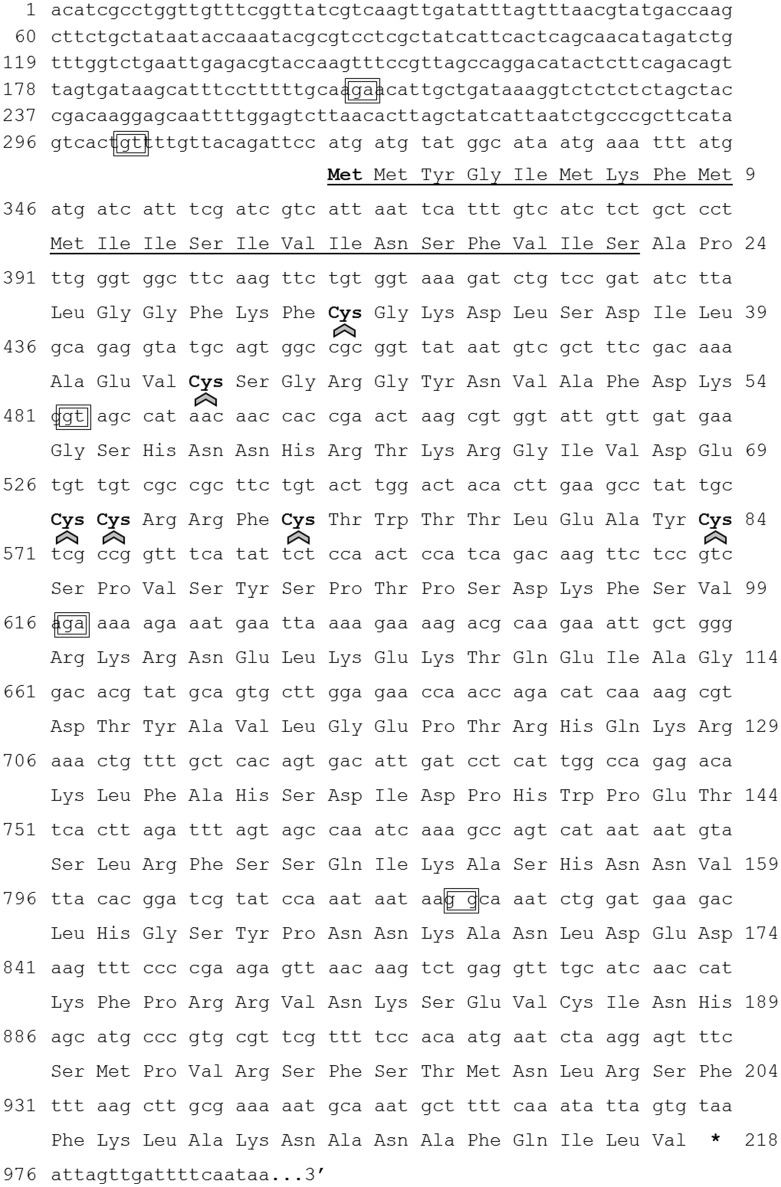
**Rhopr-IGF transcript nucleotide sequence and deduced amino acid sequence**. The numbering for the nucleotide sequence is shown at left and the numbering for amino acid sequence is shown at right. The underlined region from residue 1–22 indicates the signal peptide. The six cysteines (**Cys**) are highlighted in bold and arrows indicate the components of the three putative disulfide bonds of the mature peptide. The nucleotide pairs inside the boxes indicate the exon-exon boundaries. ^*^Indicates stop codon.

**Figure 2:**
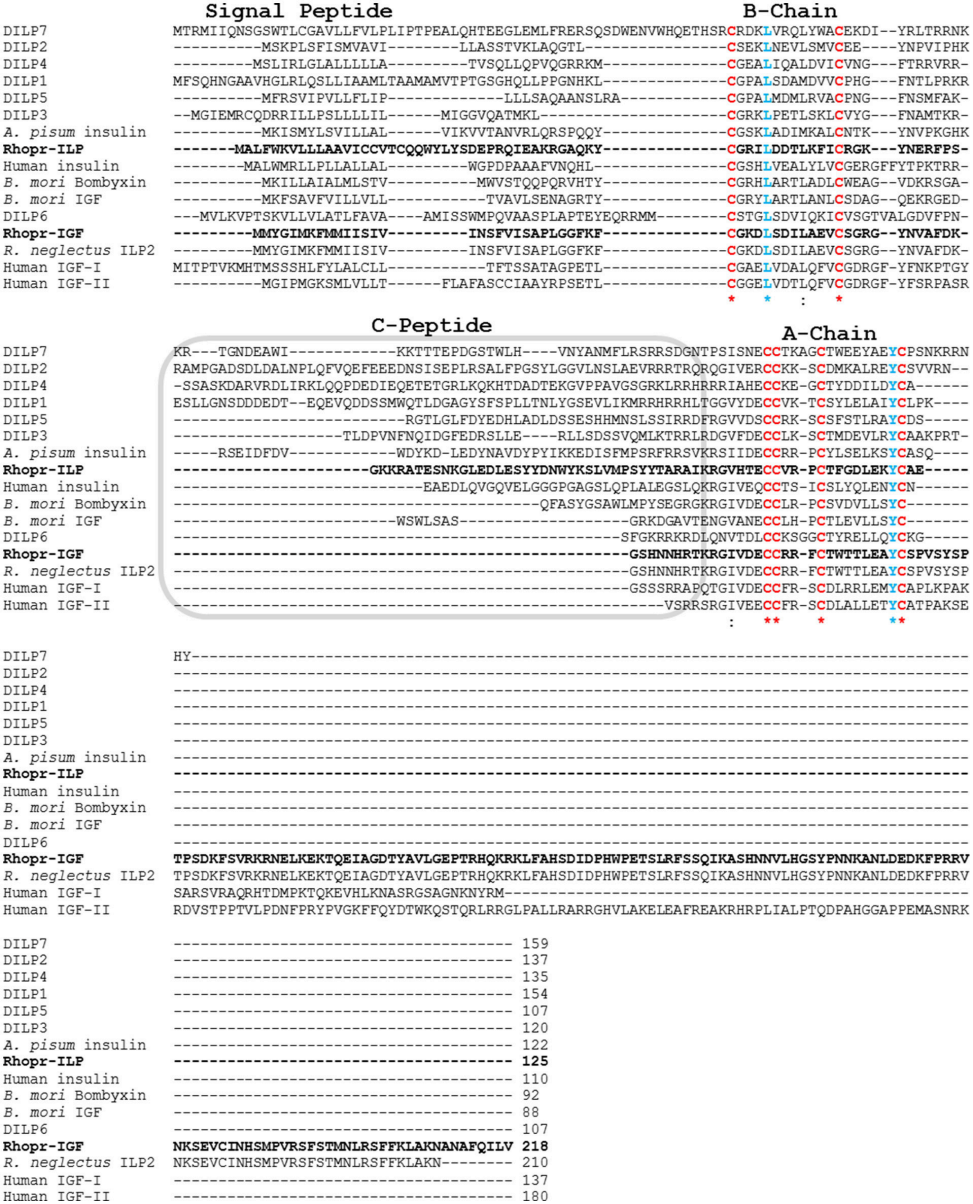
**Multiple sequence alignment of prepropeptides of insect ILPs and IGFs and human insulin and IGFs**. Rhop-IGF (KX185519) and Rhopr-ILP (KT896507) are highlighted in bold and were aligned with 11 ILPs from other insect species (*D. melanogaster* DILP1 NP_648359.1, *D. melanogaster* DILP2 NP_524012.1, *D. melanogaster* DILP3 NP_648360.2, *D. melanogaster* DILP4 NP_648361.1, *D. melanogaster* DILP5 NP_996037.1, *D. melanogaster* DILP6 *NP_570000.1, D. melanogaster* DILP7 NP_570070.1, *Acyrthosiphon pisum* insulin XP_001949438.1, *B. mori* bombyxin BAA00246.1, *B. mori* IGF NP_001138796.1, *R. neglectus* ILP2 A0A0N7Z9F4), and with human insulin and IGFs I/II (human insulin CAA23828.1, human IGF-I NP_001104754.1, human IGF-II NP_001007140.2). Chains A and B and the C-peptide region are indicated. The six conserved cysteines that form the three disulfide bonds in the mature peptides are highlighted in red, while the other highly conserved residues are highlighted in blue (L and Y), emphasized by ^*^indicates conservation between groups of strongly similar amino acids.

We identified a sequence within the *R. prolixus* genome that showed similarity to Rhopr-ILP and other insect ILPs, but also to human and insect IGFs. We have cloned and sequenced 994 base pairs of Rhopr-IGF cDNA, which has six exons, with the coding sequence starting at base 319 and ending at base 972, yielding a protein of 218 amino acids (Figure 1). The predicted preproprotein contains a signal peptide with cleavage site between residues 22 and 23, according to SignalP v4.1 and to TargetP 1.1, which also suggested that the protein is secreted to the extracellular environment. The putative Rhopr-IGF protein has six conserved cysteines as seen in other members of the insulin/IGF family that form two interchain disulfide bonds, between chains/domains A and B, and one intrachain disulfide bond, within chain/domain A (Cohick and Clemmons, 1993; Davidson, 2004), located in positions 31, 43, 70, 71, 75, and 84 (Figures 1, 2). Additionally, the predicted structure of the mature hormone resembles that of other IGFs, consisting of a single polypeptide chain stabilized by the three disulfide bonds, without the release of an internal peptide (Cohick and Clemmons, 1993). The same sequence was partially identified in a previous study, named *R. prolixus* ILP2 (Ons et al., 2011).

We have also constructed an alignment where the predicted protein sequence of Rhopr-IGF was combined with 12 ILP and IGF precursor peptides from different insects and three human insulin and IGF precursor peptides. The alignment highlights conserved residues such as the six cysteines within the A and B chains/domains, but also shows the reduced amino acid sequence between the two chains/domains in IGF sequences. Like human IGFs, Rhopr-IGF displays a longer sequence when compared to other insect ILPs, except for the partial sequence of *R. neglectus* ILP2 that shows 100% identity to Rhopr-IGF except for being shorter by eight amino acids (Figure 2).

### Spatial distribution and temporal expression of Rhopr-IGF in fifth instar *R. prolixus*

**Figure 3:**
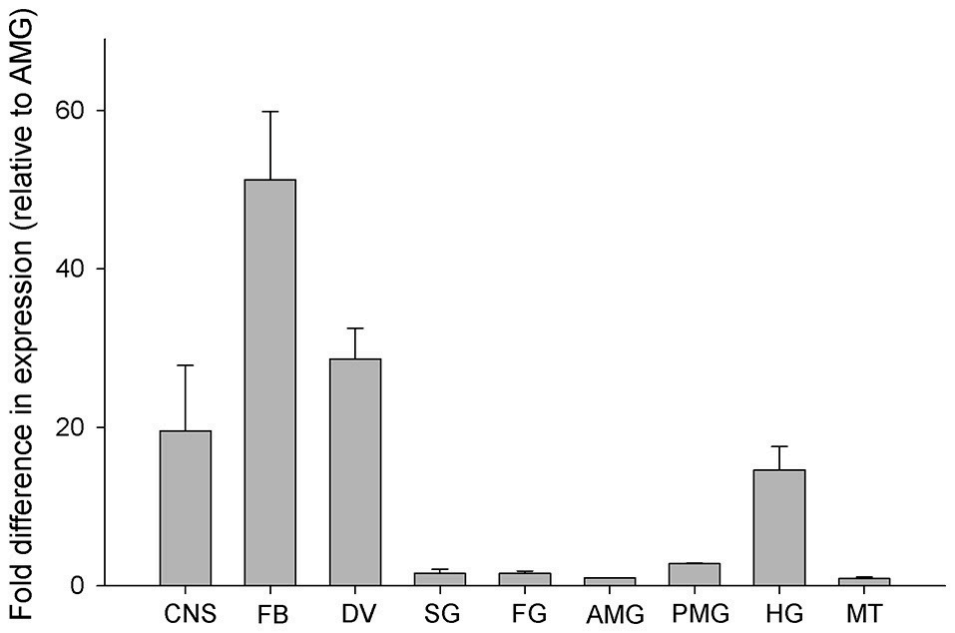
**Spatial distribution of Rhopr-IGF transcript in various tissues of fifth instar ***R. prolixus*****. Tissues were pooled together into nine different categories: central nervous system—CNS, fat body—FB, dorsal vessel—DV, salivary glands—SG, foregut—FG, anterior midgut—AMG, posterior midgut—PMG, hindgut—HG, and Malpighian tubules—MT. Quantification was performed by qPCR and fold difference in expression is relative to the anterior midgut (AMG). Results are shown as means of three biological replicates plus standard error.

Rhopr-IGF transcript expression is most abundant in the fat body, followed by the dorsal vessel, the CNS, and the hindgut, where expression ranges from 50 to 20 times higher than in the anterior midgut (AMG). The AMG was used as the reference tissue and shows a similar expression level to the other tissues tested (Figure 3).

**Figure 4:**
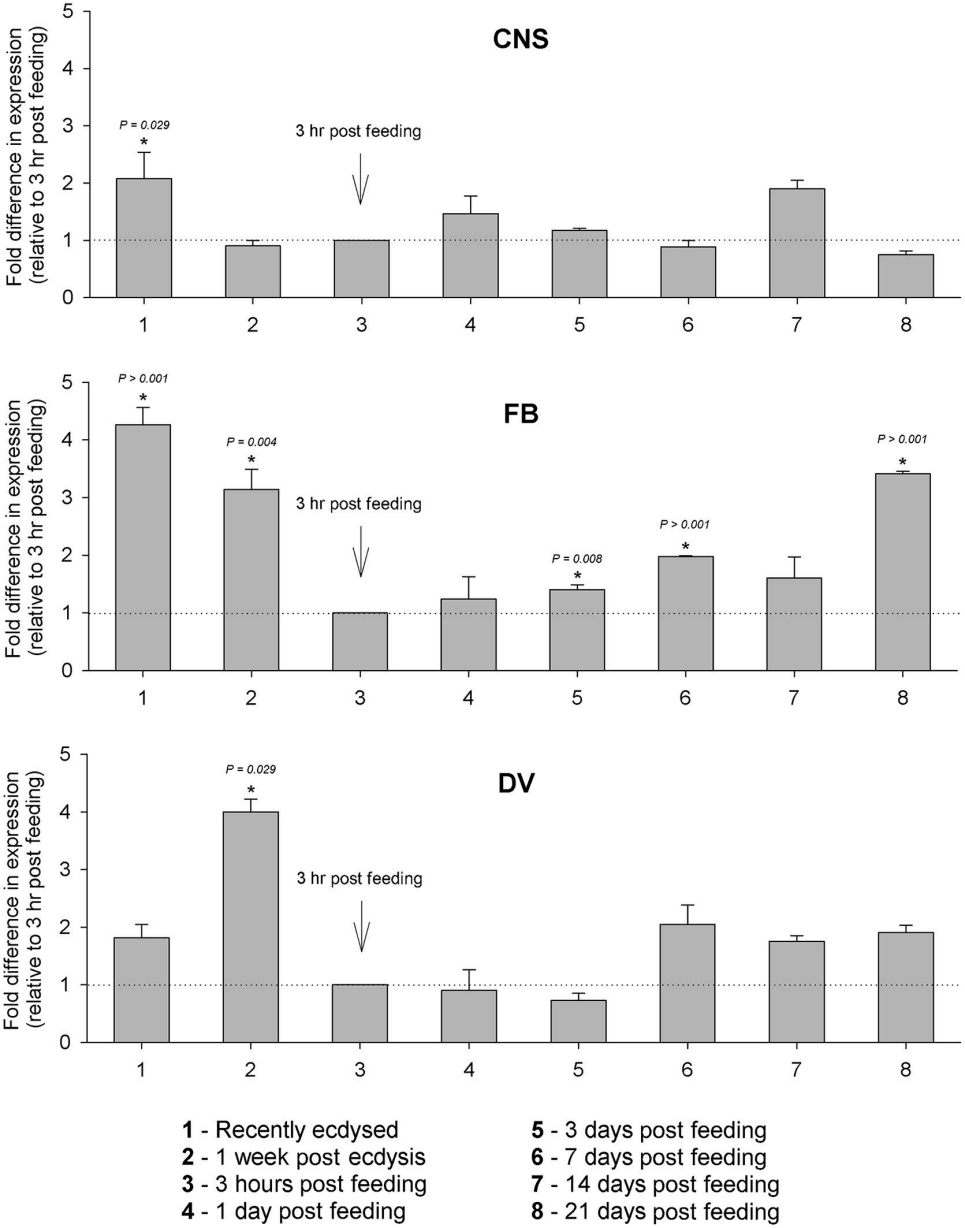
**Temporal expression of Rhopr-IGF in the CNS, fat body, and dorsal vessel of ***R. prolixus*** throughout the fifth instar**. Insects were dissected immediately and 1 week after ecdysis into fifth instars, and then 2 weeks after ecdysis one group of insects was fed on blood and then transcript expression was assayed at 3 h, 1, 3, 7, 14, and 21 days post-feeding. Five insects were dissected at each time point and the expression was quantified by real time qPCR in the CNS, the fat body, and the dorsal vessel. Results are shown as means of three biological replicates plus standard error. ^*^Indicates statistically significant differences between each time point and the time point used as reference (3 h after feeding) and the *P*-values are indicated.

Changes in Rhopr-IGF transcript expression during development were analyzed in tissues where the transcript is most abundant: the CNS, the fat body and the dorsal vessel. In the CNS, we did not observe major variations in expression throughout the different times tested, whereas in the fat body expression peaks right after the insects ecdyse into the fifth instar and before they ecdyse into the adult (21 days post-feeding). Expression in the fat body is also high 1 week after the insects ecdyse into fifths, but drops after the blood meal and stays at lower levels in the post-feeding period. In the dorsal vessel, expression peaks at 1 week after the insects ecdyse into fifths and, similar to the fat body, it drops right after the blood meal, but remains at similar levels until the last time point tested (Figure 4).

### Role of Rhopr-IGF in lipid and carbohydrate metabolism in *R. prolixus* during the post-feeding period

Injection of dsIGF reduced the expression of the transcript by 65 and 50% in the CNS, 70 and 90% in the fat body, and 40 and 70% in the dorsal vessel, at 2 and 7 days post-injection, respectively (Supplementary Figure 1). Injection of dsIGF or dsILP on transcript expression of ILP or IGF, respectively was examined and found to induce a slight compensatory upregulation but this was not significant (Supplementary Figure 2).

**Figure 5:**
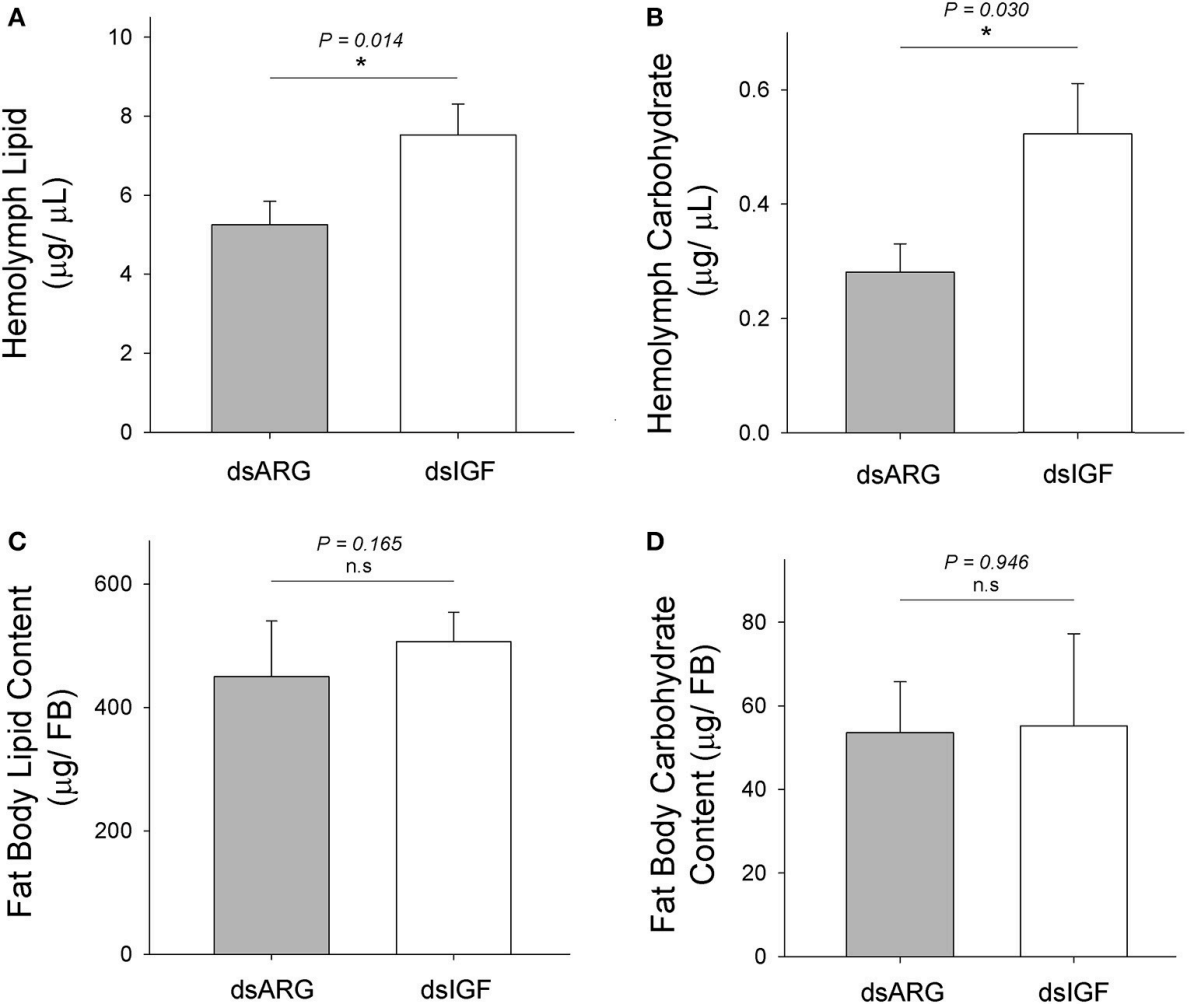
**Lipid and carbohydrate measurements in Rhopr-IGF knockdown insects**. Insects were injected with 1 μg of dsIGF or dsARG (1 week post-feeding) and the hemolymph and fat body samples were collected 1 week post injection. **(A)** Hemolymph lipid level increased after Rhopr-IGF transcript knockdown (*n* = 13 dsARG, *n* = 13 dsIGF). **(B)** Hemolymph carbohydrate level increased after Rhopr-IGF transcript knockdown (*n* = 7 dsARG, *n* = 7 dsIGF). **(C)** No statistical difference was found in fat body lipid content after Rhopr-IGF transcript knockdown (*n* = 7 dsARG, *n* = 7 dsIGF). **(D)** No statistical difference was found in fat body carbohydrate content after Rhopr-IGF transcript knockdown (*n* = 7 dsARG, *n* = 7 dsIGF). Results are shown as means plus standard errors. ^*^Indicates statistically significant differences between dsIGF and dsARG injected insects and *P*-values are indicated in the figure. n.s., denotes not significant.

At 1 week after the injection of dsIGF, hemolymph lipid, and carbohydrate levels were increased when compared to the control group (dsARG-injected) (Figures 5A,B). No significant changes in lipid or carbohydrate content of fat body were observed in Rhopr-IGF knockdown insects when compared to dsARG controls (Figures 5C,D).

### Role of Rhopr-IGF in growth of recently ecdysed adults

**Figure 6:**
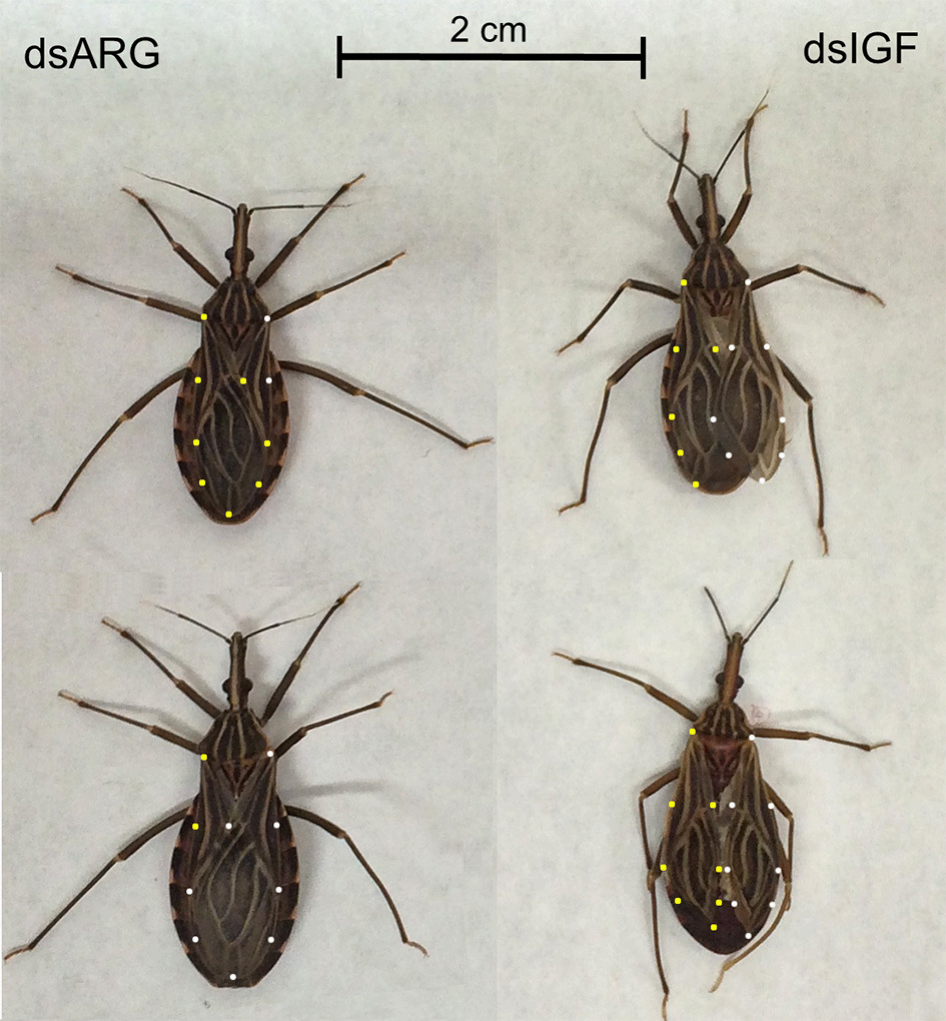
**External appearance of recently ecdysed adults after Rhopr-IGF knockdown**. Fifth instars were injected with dsARG or dsIGF 1 week after feeding and were allowed to ecdyse into adults. Pictures were taken 2–3 days after the insects ecdysed. Insects on the left were injected with dsARG and insects on the right were injected with dsIGF. Yellow dots indicate the outline of left wings and white dots indicate the outline of right wings.

**Figure 7:**
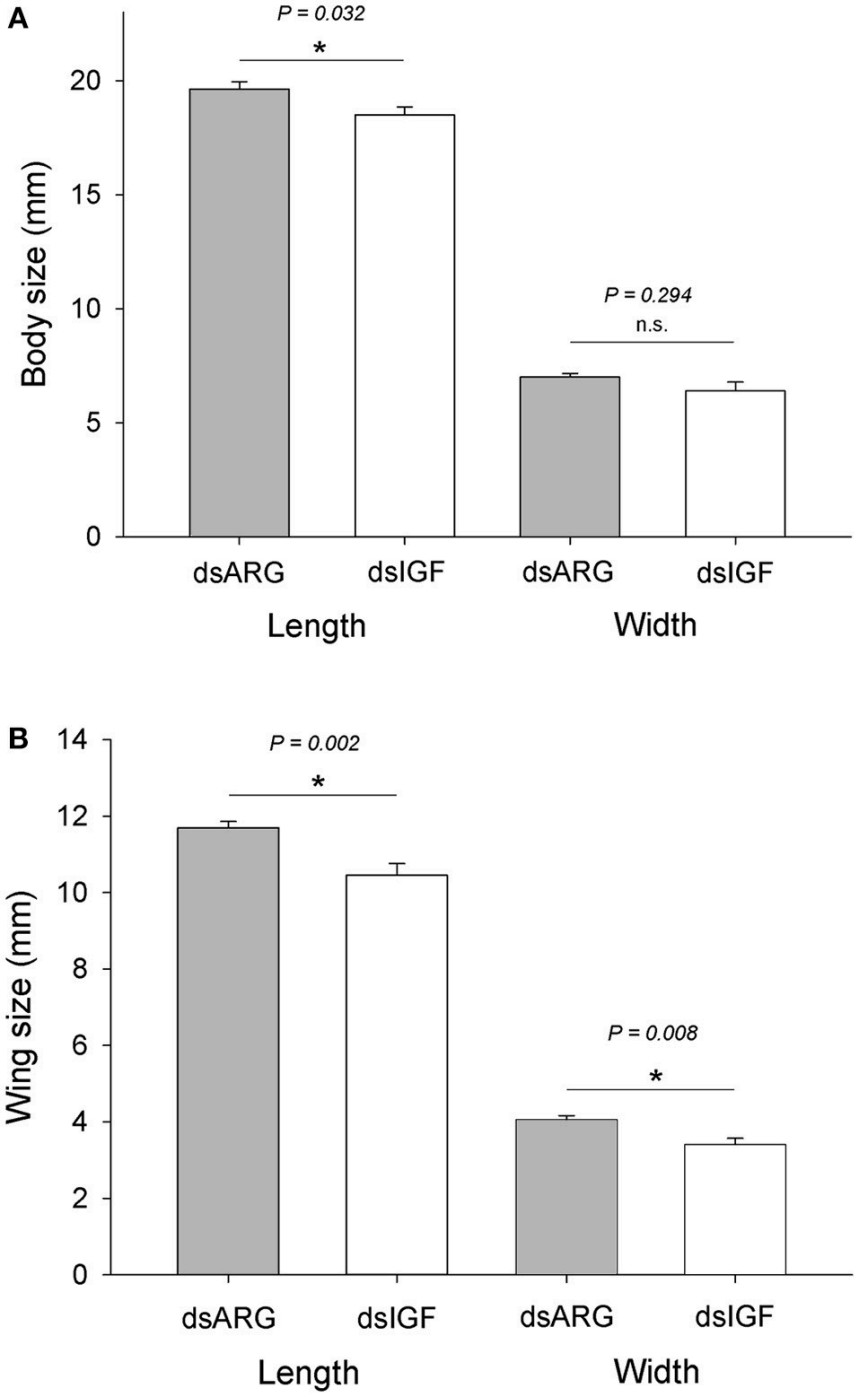
**Body and wing size in recently ecdysed adults after Rhopr-IGF knockdown**. Fifth instars were injected with dsARG or dsIGF 1 week after feeding and were allowed to ecdyse into adults. Two to three days after ecdysing the following measurements were taken: **(A)** body length and body width (*n* = 8 dsARG, *n* = 9 dsIGF), and **(B)** wing length and wing width (*n* = 16 dsARG, *n* = 18 dsIGF). Results are shown as means plus standard error. ^*^Indicates statistically significant differences between dsARG and dsIGF-injected insects and *P*-values are indicated in the figure. n.s., denotes not significant.

**Figure 8:**
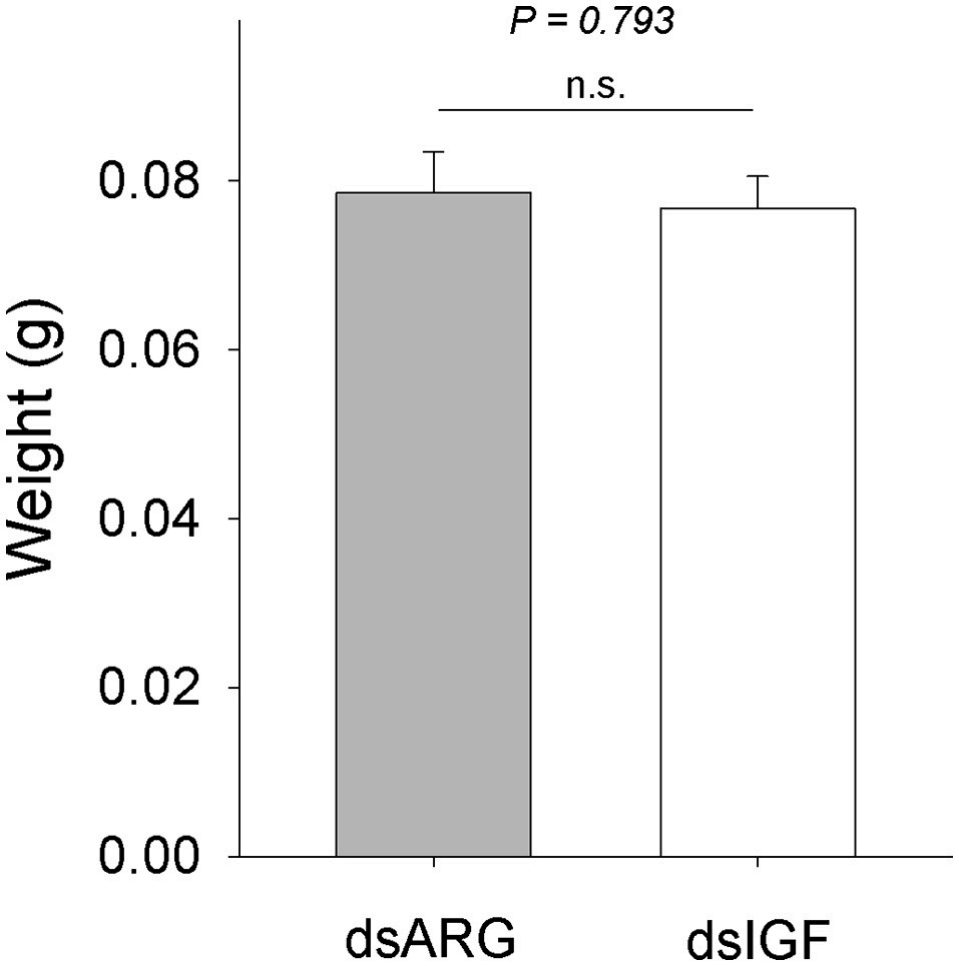
**Body weight in recently ecdysed adults after Rhopr-IGF knockdown**. Fifth instars were injected with dsARG or dsIGF 1 week after feeding and were allowed to ecdyse into adults. Two to three days after ecdysis the insects were weighed (*n* = 8 dsARG, *n* = 9 dsIGF). Results are shown as means plus standard error. The *P*-value is indicated in the figure. n.s., denotes not significant.

When fifth instar *R. prolixus* injected with dsIGF 1 week after feeding were followed through to ecdyse into adults, their external appearance was different from that of insects injected with dsARG (Figure 6). In order to quantify and assess the differences between the two groups, we weighed the insects and measured the length and width of the adult insect body and wings. Insects that had reduced Rhopr-IGF transcript expression as fifth instars, developed into adults with shorter body length along with shorter and narrower wings when compared to the dsARG controls (Figures 7A,B). On the other hand, body width and body weight did not seem to be affected by the knockdown of Rhopr-IGF (Figures 7A, 8).

## Discussion

From insects to mammals, the main effector pathway regulating growth is the insulin/IGF signaling cascade, which is modulated by nutritional conditions and developmental signals (Badisco et al., 2013; Nässel et al., 2015; Nässel and Vanden Broeck, 2016). The final body size of an adult insect is determined largely by the availability, and the proper use, of nutrients and energy in the hemolymph during the last nymphal/larval stage (Nijhout et al., 2014). In the present study, we identified an IGF that is expressed in various tissues of *R. prolixus*, but mostly in the fat body, the dorsal vessel and the CNS. *R. prolixus* only feeds once per nymphal stage when it ingests up to 10 times its own body weight in blood, and this will trigger a series of developmental changes that result in growth, development, and ultimately ecdysis into the next stage (Orchard, 2006). The mechanism that controls growth and development and therefore body size involves a variety of signaling molecules, including 20-E (Riddiford, 1993; Yamanaka et al., 2013). 20-E secretion is periodic and controls development including the production of a new cuticle beneath the old one, which is then shed off to end the process of ecdysis. Growth depends on cell division that is stimulated by the secretion of 20-E, and starts soon after the separation of the epidermis from the old cuticle (Yamanaka et al., 2013; Nijhout et al., 2014).

In fifth instar *R. prolixus*, 20-E titers increase in the hemolymph around 1 week after feeding, peak at 14–15 days, and return to basal levels around 18 days, followed by ecdysis at 20–21 days after feeding (Vafopoulou et al., 2005). The expression of Rhopr-IGF increases in the dorsal vessel and in the fat body at 1 week after feeding and in the CNS at 14 days post-feeding, which coincides with the 20-E increase and peak in the hemolymph. Subsequently, at 3 weeks after the blood meal, the expression of Rhopr-IGF peaks in the fat body, when the insects are undergoing ecdysis, suggesting that the production of Rhopr-IGF could be stimulated by 20-E. Peaks of 20-E trigger the production of IGFs by the pupal fat body of both *Drosophila* and *B. mori*, allowing tissues to properly grow during the non-feeding stage that precedes adult emergence (Okamoto et al., 2009a,b; Slaidina et al., 2009).

Around 2 weeks after the ingestion of the blood meal in *R. prolixus*, digestion is still occuring but at a much slower pace and nutrients and energy necessary for growth are being supplied by both digestion and storage tissues such as the fat body (Mariano et al., 2009). Since we observed changes in Rhopr-IGF expression during this post-feeding period, we used RNA interference to reduce the expression of the transcript and analyze the consequences on energy homeostasis and insect growth. Insects were injected with double-stranded RNA targeting the Rhopr-IGF transcript at 1 week after feeding, and 1 week later, fat body and hemolymph were collected for measuring lipids and carbohydrates. Insects that had the Rhopr-IGF transcript expression reduced showed increased lipid and carbohydrate hemolymph levels, but no changes in fat body lipid or carbohydrate content when compared to control insects. These results suggest that the knockdown of Rhopr-IGF may change how tissues demand/use energy sources that are present in the hemolymph and that have been acquired through digestion. The knockdown may not affect the process of energy storage in the fat body. Although there may be some compensatory up-regulation of Rhopr-ILP transcript, we did not find this to be statistically-significant.

The insulin signaling process is mediated by both insulin and IGF, where insulin controls lipid and carbohydrate metabolism, and IGF regulates growth through cell division (Thissen et al., 1994; Saltiel and Kahn, 2001). We have recently shown that Rhopr-ILP, which is only expressed in median neurosecretory cells in the brain and is similar to human insulin, is involved in the regulation of lipid and carbohydrate homeostasis in the hemolymph and lipid and carbohydrate storage in the fat body (Defferrari et al., 2016). Therefore, Rhopr-ILP and Rhopr-IGF work together to regulate nutrient storage and homeostasis, as well as, growth and development. It has been suggested (Ons et al., 2011) that *R. prolixus* possesses two other ILPs (Rhopr-ILP3 and Rhopr-ILP6). We have so far been unable to clone these or to confirm their identities through bioinformatics of the *R. prolixus* genome. The number of ILP genes varies considerably between insect species, with 38 present in *B. mori*, 8 in *Drosophila* and 1 in *Schistocerca gragaria* (Nässel et al., 2015), and so *R.prolixus* appears to be at the lower end of this range.

As discussed by Lavine et al. (2015) the ILP/IGF pathway regulates growth by integrating physiological conditions and metabolism in a condition-dependent manner. In *Drosophila* the IGF, DILP6, promotes growth in wandering larvae and pupae, but not in early larvae (Slaidina et al., 2009). Interestingly, though, the levels of hemolymph trehalose, glucose, and triacylglycerol were not altered in DILP6 mutant larvae at the wandering stage suggesting that in *Drosophila*, and at this stage, DILP6 is not required for metabolic regulation (Okamoto et al., 2009b; Slaidina et al., 2009). These differences from *R. prolixus* emphasizes the stage, condition, and species-dependent manner of ILP/IGF signaling.

Since growth relies on the ability of cells to access and assimilate nutrients and the reduced expression of Rhopr-IGF most likely impairs that ability, we followed dsIGF knockdown insects through ecdysis into adults to evaluate the role of Rhopr-IGF in development. We observed that, after ecdysis, adults with reduced Rhopr-IGF transcript levels as fifth instars displayed a shorter body length along with shorter and narrower wings when compared to control insects, but showed no differences in body width or body weight. *R. prolixus* is a hemimetabolous insect, meaning that after five nymphal stages it will ecdyse into an adult without undergoing a pupal stage. In such insects, growth of appendages happens gradually along with the body during each molt cycle for nymphal stages, except for the wings and reproductive organs that only grow during the last instar before they ecdyse into adults (Nijhout et al., 2014). In *Drosophila*, the knockdown of DILP6 reduces the amount of energy used to build adult tissues during the pupal stage, resulting in adults with smaller bodies when compared to controls (Slaidina et al., 2009). Analysis of DILP6 mutants showed that they had ~10% reduced final adult body size which was not related to cell size but was due to a reduction in cell number (Okamoto et al., 2009b).

We also observed that the expression of Rhopr-IGF is increased in the CNS, the fat body, and the dorsal vessel, after the insects ecdyse into fifth instars. One week after ecdysis, the fat body and the dorsal vessel still show high transcript expression levels, suggesting that besides being involved in growth, Rhopr-IGF might have a role during the non-feeding stage that precedes the blood meal. It has been shown in *Drosophila* that the insulin signaling pathway is essential for starvation resistance, and that DILP6 is required for the accumulation of lipids in the fat body in order to maintain the lipid reserves that allow the flies to tolerate periods of starvation (Chatterjee et al., 2014). We have previously shown that Rhopr-ILP is necessary for energy homeostasis in unfed fifth instars, and insects lacking Rhopr-ILP have increased lipid and carbohydrate levels in the hemolymph along with increased lipid content and decreased carbohydrate content in the fat body (Defferrari et al., 2016). Further, studies are necessary in order to investigate how Rhopr-IGF affects energy metabolism during unfed stages and how it interacts with Rhopr-ILP signaling. Nonetheless, the results presented here indicate that Rhopr-IGF most likely plays multiple roles during different stages of nutrition in *R. prolixus*, regulating growth and modulating the utilization of hemolymph lipid and carbohydrate in the post-feeding period.

## Author contributions

MD designed the study, conducted the experiments, and wrote the manuscript, IO and AL helped in the design of the study, revised the manuscript, and supervised the work.

### Conflict of interest statement

The authors declare that the research was conducted in the absence of any commercial or financial relationships that could be construed as a potential conflict of interest.

## Supplementary material

Supplementary Figure 1 **Rhorp-IGF expression analysis in fifth instar ***R. prolixus*** injected with dsRNA**. Insects were injected with dsARG or dsIGF and the CNS, fat body, and dorsal vessel were dissected 2 and 7 days after the injections. Rhopr-IGF expression was quantified using qPCR, where the expression in dsIGF-injected insects was quantified relative to the expression in dsARG-injected insects using the ΔΔCt method (Pfaffl, 2001). Results are shown as means of three biological replicates plus standard error.

Supplementary Figure 2 **(A)** Rhopr-IGF expression after Rhopr-ILP knockdown; **(B)** Rhopr-ILP expression after Rhopr-IGF knockdown. Expression was measured 7 days after the injection of the dsRNAs. Results are shown as means plus standard error. The *P*-value is indicated in the figure. n.s., denotes not significant.

Supplementary Table 1 **Gene specific primers used for performing RACE PCR of both 5′ and 3′ of Rhopr-IGF, for the amplification of open reading frame, for q PCR, and for the construction of the dsRNA used for Rhopr-IGF expression knockdown**.
