# Supplementary material for: An Insulin-Like Growth Factor in Rhodnius prolixus Is Involved in Post-feeding Nutrient Balance and Growth
Source: Front Neurosci. 2016 Dec 9;10:566. doi: 10.3389/fnins.2016.00566 (PMC5145886; doi:10.3389/fnins.2016.00566)
Supplement: Supplementary Table 1 — Gene specific primers used for performing RACE PCR of both 5′ and 3′ of Rhopr-IGF, for the amplification of open reading frame, for q PCR, and for the construction of the dsRNA used for Rhopr-IGF expression knockdown. [file Table1.PDF]

Supplementary Table 1. Gene specific primers used for performing RACE PCR of both 5’ and 3’ of Rhopr-IGF, for the amplification of open reading frame, for q PCR, and for the construction of the dsRNA used for Rhopr-IGF expression knockdown.

**RACE PCR primers (5’-3’)**	
IGF RACE Fw1	CGCCGGTTTCATATTCTCC
IGF RACE Fw2	AGATTTAGTAGCCAAATCAAAGCC
IGF RACE Rv1	TTGTTATGGCTACCTTTGTCG
IGF RACE Rv2	AACTTGAAGCCACCCAAAGG
**Primers for amplification of complete sequence (5’-3’)**	
IGF Fw	ACATCGCCTGGTTGTTTCG
IGF Rv	TTATTGAAAATCAACTAATTTACAC
**Quantitative PCR primers (5’-3’)**	
IGF qPCR Fw	TGTCATCTCTGCTCCTTTGG
IGF qPCR Rv	TTGTTATGGCTACCTTTGTCG
**dsRNA primers for RNAi (5’-3’)**	
IGF dsRNA Fw	CGCTTCATAGTCACTG
IGF dsRNA Rv	AAACTTGTCTTCATCCAGATTTGCC
IGF dsRNA Fw T7	**TAATACGACTCACTATAGGGAGA** CGCTTCATAGTCACTG
IGF dsRNA Rv T7	**TAATACGACTCACTATAGGGAGA** AAACTTGTCTTCATCCAGATTTGCC

* **TAATACGACTCACTATAGGGAGA** = T7 RNA polymerase promoter region
